# Cardiometabolic Risk Factors among Healthcare Workers: A Cross-Sectional Study at the Sefwi-Wiawso Municipal Hospital, Ghana

**DOI:** 10.1155/2018/8904548

**Published:** 2018-04-23

**Authors:** James Osei-Yeboah, Kenneth Kwame Kye-Amoah, William K. B. A. Owiredu, Sylvester Yao Lokpo, Joseph Esson, Beatrice Bella Johnson, Paul Amoah, Romeo Asumbasiya Aduko

**Affiliations:** ^1^Department of Medical Laboratory Sciences, School of Allied Health Sciences, University of Health and Allied Sciences, Ho, Ghana; ^2^Laboratory Department, Sefwi-Wiawso Municipal Hospital, Ghana Health Service, Sefwi-Wiawso, Western Region, Ghana; ^3^Department of Molecular Medicine, School of Medical Sciences, Kwame Nkrumah University of Science and Technology, Kumasi, Ghana; ^4^Department of Clinical Biochemistry, Diagnostic Directorate, Komfo Anokye Teaching Hospital, Kumasi, Ghana; ^5^Department of Nursing, School of Nursing and Midwifery, University of Health and Allied Sciences, Ho, Ghana; ^6^Clinical Biochemistry Unit, Laboratory Department, Volta Regional Hospital, Ghana Health Service, Ho, Volta Region, Ghana

## Abstract

**Background:**

There is a dearth of information about the burden of cardiometabolic risk factors among the Ghanaian health workforce in the Western Region. This study sought to determine the prevalence of cardiometabolic risk factors among healthcare workers at the Sefwi-Wiawso Municipal Hospital in the Western Region of Ghana.

**Materials and Methods:**

A hospital-based cross-sectional study involving 112 employees of the Sefwi-Wiawso Municipal Hospital was conducted. The cardiometabolic risk variables assessed were obesity, hypertension, dyslipidaemia, and diabetes. Sociodemographic parameters were also captured. The prevalence of hypertension and obesity was determined using the JNC VII panel and WHO BMI criteria for obesity classifications. Blood lipids and glucose concentrations were evaluated using standard methods.

**Results:**

The prevalence of hypertension and prehypertension was 16.07% and 52.68%, respectively. About 38.39% of participants were overweight, and 12.50% were obese. Atherogenic dyslipidaemia was 26.79%, whereas prediabetes glycaemic levels and diabetes incidence were 5.41% and 4.50%, respectively. Fifty percent (50.00%) of participants presented at least one cardiometabolic risk factor. Aging and adiposity were associated with increasing cardiometabolic risk.

**Conclusion:**

Cardiometabolic risk factors are prevalent among healthcare providers in Sefwi-Wiawso. The cardiometabolic dysregulation observed among this cohort of healthcare professionals may be modulated by age and adiposity.

## 1. Introduction

Healthcare workers are an essential group of professionals whose work is critical to the maintenance of a healthy society [1]. Healthcare providers primarily comprise certified medical personnel who are mainly physicians, nursing staff, medical scientists, pharmacist, and medical technicians as well as nonclinical support staff including the administrative class [2]. Owing to their specialised training, healthcare professionals are expected to demonstrate a high knowledge and awareness regarding health consequences of lifestyle changes such as diabetes and cardiovascular diseases [3]. Considering the health-related knowledge at the disposal of hospital workers and their proximity to healthcare delivery, the assumption will be that the prevalence of cardiometabolic diseases and its modifiable risk factors would be relatively low [1, 4]. However, certain work-related risk factors, such as shift work and mental and physical stress, which characterize the work environment of the hospital put health workers among a high occupational risk group for certain disease conditions [5].

Healthcare workers are mentors to the general population for a healthy life and have the principal responsibility of encouraging appropriate lifestyle changes that affect the prevention of these diseases [1, 6]. Evidence suggests that there is a strong and consistent relationship between healthcare workers choices and the recommendations he or she makes to his or her patients [7]. Thus, preventing cardiovascular disease and other related metabolic risk factors among healthcare workers is a major strategy to achieving a healthy workforce in the workplace as well as in the general population [8].

Comprehensive information on the prevalence and character of cardiometabolic risk factors among healthcare professionals is essential to inform design and implementation of interventions to reduce cardiometabolic risk among healthcare workers [9]. To our knowledge, no work assessing the cardiometabolic risk profiles of Ghanaian healthcare workers in the Western Region has been conducted. In the light of this knowledge gap, the current study, therefore, sought to evaluate the prevalence of cardiometabolic risk factors among employees of the Sefwi-Wiawso Municipal Hospital in the Western Region of Ghana.

## 2. Materials and Methods

### 2.1. The Study Site

The Municipal Hospital serves as a referral facility for the various Health Centers, Community-based Health Planning and Services (CHPS) compounds, and Clinics within the Sefwi-Wiawso Municipality and parts of the Sefwi Akontombra, Sefwi Juaboso, and Bibiani Anhwiaso Bekwai districts. The total bed capacity of the hospital is 94. The facility operates an outpatient department (OPD) with an average attendance of 150 clients per day. The OPD consists of an emergency unit with four beds, triaging area, antenatal clinic (ANC), antiretroviral (ART) clinic, tuberculosis (TB) clinic, pharmacy, laboratory including blood bank services, ear, nose and throat (ENT) clinic, dental clinic, and an eye clinic. The inpatients are managed in four wards, namely, children's ward, female ward, maternity ward, and males ward. At the time of this study, the staff strength stood at 224 employees including clinical and nonclinical staff.

### 2.2. Study Design and Study Population

This hospital-based cross-sectional study which involved 112 hospital employees participating in an annual medical screening program at the Sefwi-Wiawso Municipal Hospital in the period between 11 and 13 May 2016. The study included 48 male and 64 female participants between ages of 22 and 59 years.

### 2.3. Sample Size

Using the Raosoft online sample size calculator (http://www.raosoft.com/samplesize.html), the recommended minimum sample of 105 participants was calculated at 95% confidence level, 7% margin of error, and a response distribution of 50%.

### 2.4. Blood Pressure (BP) Measurement

The blood pressure (BP) measurements were taken using the OMRON digital fully automated blood pressure monitor (OMRON Healthcare, IntelliSense BP785, HEM-7222). After participants had rested in a sitting position for at least ten minutes by qualified nurses, two measurements were taken with an appropriately sized cuff at a one-minute interval on the right arm, with the arm supported at heart level and feet flat on the floor [10]. The Seventh Report of the Joint National Committee on Prevention, Detection, Evaluation, and Treatment of High Blood Pressure (JNC VII) criteria for the classification of blood pressure was used. Normotensives were classified as systolic blood pressure (SBP) < 120 mmHg and diastolic blood pressure (DBP) < 80 mmHg, prehypertension (SBP 120–129 mmHg or DBP 80–89 mmHg), hypertension stage 1 (SBP 140–159 mmHg or DBP 90–99 mmHg), and hypertension stage 2 (SBP ≥ 160 mmHg or DBP ≥ 100 mmHg) [11].

### 2.5. Anthropometric Measurement

A dual-purpose weight and height scale was used to measure body weight of participants to the nearest 0.1 kg and height to the nearest 0.1 cm with participants standing upright in a relaxed posture, heels together, feet slightly spread without shoes, and in light clothing (Health O meter® Professional 104 9500 West 55th St. McCook, IL 60525-7110, USA). Body mass index (BMI) was calculated by dividing weight (kg) by the square of the height in meters (m^2^). Using the WHO classification of obesity, underweight was defined as BMI < 18.5 kg/m^2^, overweight as BMI ≥ 25.00 kg/m^2^, obesity class I as BMI 30.00–34.99 kg/m^2^, obesity class II as BMI 35.00–39.99 kg/m^2^, and obesity class III as ≥40 kg/m^2^ [12].

### 2.6. Biochemical Assays

Following an overnight fast, about 5 ml venous blood sample was drawn from the antecubital vein between 7 a.m. and 10 a.m., of which 3 milliliters was dispensed into a vacutainer® serum separator tube using the closed vacutainer system and 2 ml into fluoride oxalate tubes. Whole blood samples were centrifuged at 2500 rpm for 5 minutes to obtain plasma, and serum after samples was allowed to clot. Using serum and plasma, the following biochemical assays were estimated: fasting blood glucose (FBG), total cholesterol (TC), triglyceride (TG), and high-density lipoprotein cholesterol (HDL-C). Very low-density lipoprotein cholesterol (VLDL-C) and Low-density lipoprotein cholesterol (LDL-C) were calculated using the Frederickson-Friedewald's formula where LDL-C = TC − HDL-C − TG/2.2 [13]. The methods adopted for various assays were predetermined by the reagent manufacturer (ELITech Clinical Systems, SAS, Zone Industrielle-61500 SEES, France). All biochemistry assays were carried out at the Sefwi-Wiawso Municipal Hospital laboratory using fully automated Selectra Pro S Chemistry autoanalyzer (Vital Scientific, B.V. Kanaalweg 24, Netherlands).

### 2.7. Ethical Consideration

Written approval for the study was sought and obtained from the management of Sefwi-Wiawso Municipal Hospital. Participation was voluntary, and all participants consented to participate in the study. Analysis of the data was anonymous and nonlinked, and no participant names were used.

### 2.8. Statistical Analysis

All continuous variables were tested for normality using the Kolmogorov-Smirnov normality test with Lilliefors Significance Correction. Continuous parametric variables were expressed as a mean ± standard deviation, and nonparametric variables were expressed as median (minimum and maximum). Categorical variables were expressed as frequencies and proportions. Comparisons of parameters were performed using unpaired *t*-tests, Mann–Whitney *U* test, Chi-square (*χ*2) tests, or Fisher exact tests where appropriate. A *p* < 0.05 was considered as statistically significant for all analyses. IBM Statistical Package for the Social Sciences version 22.00 was used for data analysis (SPSS Inc., Chicago, USA; https://www.spss.com).

## 3. Results

The study involved 112 health workers with 64 (57.14%) being female and the rest males 48 (42.86%). The average age of the participants in this study was 32.1 ± 8.9 ranging from 22 years to 59 years with majority of 40 years and below (86.61%). The male and female health workers statistically presented with comparable age 31.9 ± 7.5 (*p* = 0.8462). The average systolic and diastolic blood pressure were 117.7 and 76.7 mmHg, respectively. The averaged body mass index of the female participant was 26.3 Kg/m^2^ and was significantly higher than that of the male participants 23.9 Kg/m^2^ (*p* = 0.0019). As seen from Table 1, there was no gender variation among the study participants in the atherogenic and glycaemic variable measured. (See Table 1).

Using the JNC VII criteria for the classification of blood pressure, 18 (16.07%) and 59 (52.68%) of the health workers presented with hypertension and prehypertension, respectively. Among those presenting with hypertension, 15 (83.33%) were classified as stage 1 and the remaining 3 (16.67%) were in stage 2. Also among the health workers classified as hypertensive, 8 (44.44%) presented with both high systolic and diastolic blood pressure, 9 (50.00%) presented with isolated high diastolic blood pressure, and 1 (5.56%) presented with isolated high systolic blood pressure (See Table 2).

Using the WHO BMI classification for obesity, 2 (1.79%) of the participants were classified as underweight, 43 (38.39%) were overweight, and 14 (12.50%) were obese. Among the obese participants, 78.57% were classified as class I and 21.43% as class II obesity. Obesity was significantly higher among the female health workers, 13 (20.31%), than their male counterparts, 1 (2.08%). Participants who presented with atherogenic dyslipidaemia were 18.75% with raised total cholesterol (TC), 10.74% with raised triglyceride (TG), and 3.57% with raised very low-density lipoprotein (VLDL). Dyslipidaemia was comparable across gender. Five persons (4.46%) had diabetes, and six (5.36%) had prediabetic glycaemic levels. The difference in glycaemic levels did not significantly vary by gender (see Table 3).

Hypertension was found to be 33.33% among participants older than 40 years and estimate as between 13% and 14% among those below 40 years. Diabetes peaked among participants above 40 year to 50 year old. In general, high prevalence of obesity and dyslipaemia was observed among participants above 30 years (see Table 4).

**Figure 1:**
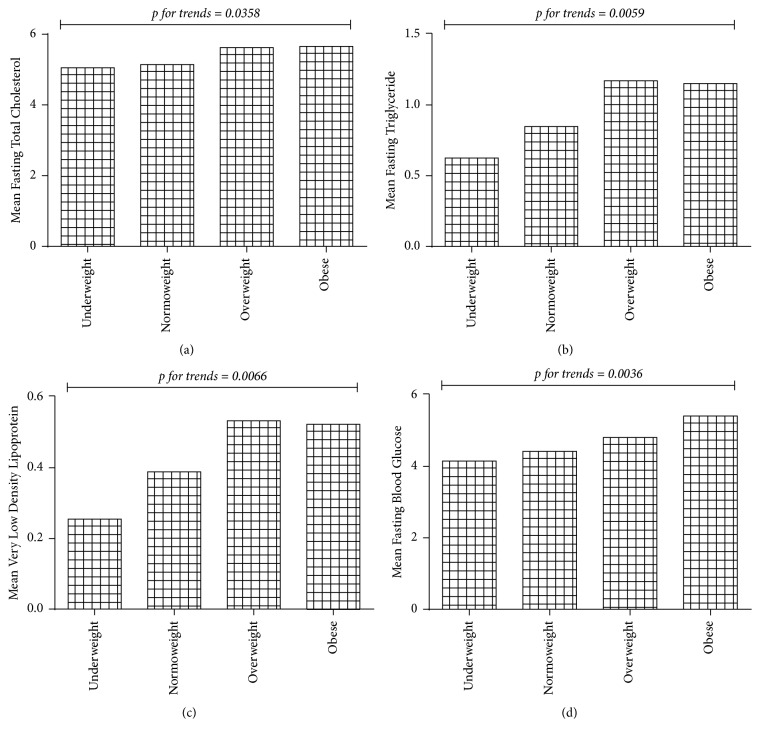
Association between adiposity, atherogenic indices, and glycaemia among health workers in Sefwi-Wiawso Municipal Hospital.

A significant additive trend was observed between the mean atherogenic dyslipidaemia markers measured and adiposity grading. In all cases, the levels of atherogenic dyslipidaemia markers increased from underweight and peaked at overweight or obese (Figures 1(a), 1(b), and 1(c)). Glycaemia levels showed a significant trend across increasing adiposity grading, troughed among those graded as underweight and peaked among those graded as obese (Figure 1(d)) (see Figure 1).

In general, aging was associated with increasing blood pressure, glycaemia, and dyslipidaemia. Adiposity was positively correlated with glycaemic levels and dyslipidaemia. Increasing levels of glycaemia were associated with corresponding increases in systolic blood pressure and dyslipidaemia (see Table 5).

## 4. Discussion

The current study included 112 fairly young (32.1 ± 8.9 years) healthcare professionals employed at the Sefwi-Wiawso District Hospital in the Western Region of Ghana at the time of the study. The demographic characteristics of the study population (see Table 1) are comparable to reports of healthcare professionals in other facilities, Kadjebi Ghana [14] and Lagos Nigeria [15]. The similarities include age and gender distribution.

Using the WHO BMI classification for obesity, 43 (38.39%) of the participants were overweight, and 14 (12.50%) were obese. The reported burden of overweight and obesity among health care givers varies across various jurisdictions. At the Kadjebi District in the Volta Region of Ghana, a lower percentage of overweight (25%) and a similar percentage of obesity (12.7%) were recorded. [14]. Duodu et al. [16] observed that 31.8% of healthcare professional in the Hohoe Municipal Hospital were overweight and 28.9% were obese. Among a tertiary health institution in Accra, Aryeetey and Ansong [17] found that 43% of the employees were overweight and 13% were obese. In other African countries, overweight and obesity among healthcare employees ranged from 31.4 to 44.7% and 23.25 to 27.35% in Nigeria [4, 15, 18], Skaal and Pengpid [19] reported 26.5% and 47% (overweight and obesity) in South Africa, and Ondicho et al. [9] reported 30.9% and 27.9% in Kenya.

Gender preponderance to obesity among the African population in general and healthcare employees is tilted toward females [9, 15, 18]. The suggested reasons for this phenomenon include the relatively sedentary lifestyle of African women, cultural appreciation of fatness as beauty, a sign of affluence and good living, and genetics [19, 20]. In concordance with earlier reports, the proportion of female healthcare workers, 13 (20.31%), with obesity in the current study was significantly higher than their male counterparts, 1 (2.08%). The burden of overweight and obesity is linked to noncommunicable diseases (NCDs) such as diabetes, hypertension, cardiovascular disorders, and cancers, all of which could increase morbidity and mortality [15, 21–23]. In the workplace, obesity is reportedly associated with weight discrimination, increased rates of absenteeism, presenteeism (health-related limitations at work), occupational injury, short-term disability, and reduced productivity [24–27]. Moreover, obesity is related to early retirement from the workplace, and this could lead to difficulty in retaining health workers whose numbers are already low in most developing countries [28].

In the current study, using the JNC VII criteria for the classification of blood pressure, 18 (16.07%) and 59 (52.68%) of the health workers presented with hypertension and prehypertension, respectively. Among those presenting with hypertension, 15 people representing 83.33% were classified as stage 1, and the remaining (16.67%) were in stage 2. Additionally, among the hypertensive healthcare workers, 8 (44.44%) presented with both high systolic and diastolic blood pressure, whereas 9 (50.00%) presented with isolated high diastolic blood pressure and 1 (5.56%) presented with isolated high systolic blood pressure (Table 2). The prevalence of hypertension (16.07%) recorded in this study is higher than the 5.7% reported by Kasu et al. [14] and 14.1% by Duodu et al. [16] among Ghanaian healthcare givers. However, the rate is lower than the 17.5% to 37.5% recorded among health workers in a systematic review of hypertension prevalence in West Africa as well as the 34% prevalence reported among College of Health Sciences employees in a Ghanaian University [17, 29]. Moreover, prevalence rates higher than that of the current study have also been reported in Nigerians [30], Indians [3], and Europeans [5]. Of particular interest in the current study is the high rate of prehypertension observed among this apparently healthy Ghanaian health work professionals. Prehypertension may offer a window of opportunity to prevent established hypertension from developing; however, when left unmanaged, it could lead to increases in hypertension-related cardiovascular burden [31]. A previous report by Jardim et al. [32] in a prospective cohort study among a group of healthcare professionals observed an increase in the prevalence of hypertension from 6.0% to 16.7% after 15 years of follow-up.

Disturbances in glucose and lipid metabolism are often contemporaneous and strongly linked to subsequent development of cardiovascular morbidity in a state of insulin resistance [33]. The prevalence of undiagnosed diabetes among the study population was 5 (4.50%), and 6 (5.41%) presented with prediabetic glycaemic levels. Thirty (26.29%) of the healthcare providers presented with atherogenic dyslipidaemia, with 5 (16.67%) of these participants presenting with multiple atherogenic lipid abnormalities. Significantly, all the employees with diabetes were clinical staff. Also a greater proportion of the clinical staff presented with raised TCh (23.26%) levels compared to their nonclinical counterparts (3.85). Consistent with the findings of the current study, earlier investigators have reported the incidence of dyslipidaemia and disordered glucose metabolism among Asian healthcare providers [2, 3, 33].

We observed that an increasing level of glycaemia was associated with a corresponding increase in atherogenic lipid parameters (see Table 5). Though, not clearly understood, postulated mechanisms have linked glycaemic dysregulation and dyslipidaemia. In insulin-resistant states, there is upregulation of insulin levels, increased hepatic gluconeogenesis and glucose output, reduced suppression of lipolysis leading to a high free fatty acid influx, and increased hepatic VLDL secretion resulting in hypertriglyceridaemia and reduced plasma levels of HDL [34].

A significant additive trend was observed between the mean atherogenic dyslipidaemia markers and adiposity grading. In all cases, the levels of atherogenic dyslipidaemia markers increased from underweight and peaked at overweight or obese (Figures 1(a), 1(b), and 1(c)). Similarly, glycaemia levels showed a significant trend across increasing adiposity grading, troughing among those graded as underweight, and peaking among those graded as obese (Figure 1(d)). Though the precise mechanisms are still unclear, accumulating evidence indicates that obesity is closely associated with an increased risk of metabolic diseases such as insulin resistance, type 2 diabetes, and dyslipidaemia [35]. Evidence suggests that chronic inflammation in the adipose tissue may play a critical role in the development of obesity-related metabolic dysfunction [36].

Aging has been demonstrated to be a significant determinant for the development of morbid heart conditions and insulin resistance states leading to type 2 diabetes [37, 38]. Though in the current study increasing age was found to associate with most cardiometabolic parameters assessed (see Table 5), the age stratification of cardiometabolic risk factors revealed a relatively high levels of hypertension (13% to 14%), obesity (7% to 21%), and dyslipidaemia (16% to 38%) among respondents 40 years and younger (see Table 4). Among black populations, relatively early onset of cardiovascular abnormalities has been reported [39–41]. Owusu et al. [42] postulated the critical age threshold of 39 years for hypertension among a Ghanaian population. Though genetic predisposition to this phenomenon has been suggested, there is a consensus that this is modulated by environmental factors [43, 44]. The main attributed factor for the surge in cardiometabolic diseases in the Ghanaian population is urbanization, which is accompanied with lifestyle modifications such as diet change, physical inactivity, stress, and risk behaviours (smoking, alcohol consumption, etc.) [42, 45, 46].

The hypothesis that, armed with the knowledge of the prevention of cardiometabolic risk factors, healthcare workers were expected to present lesser prevalence of risk factors to the general population did not hold in this study, since 56 (50.0%) of this study population presented with at least one of the cardiometabolic risk factors assessed (obesity, dyslipidaemia, hypertension, and diabetes), 47 (42.0%) presented with one risk factor, 8 (7.1%) presented with two risk factors, and 1 (0.9%) presented with four cardiometabolic risk factors.

## 5. Conclusion

Cardiometabolic risk factors are prevalent among healthcare providers in Sefwi-Wiawso Municipal Hospital. The cardiometabolic dysregulation observed among this cohort of healthcare professionals may be modulated by age and adiposity. Lifestyle measures to reduce the burden of cardiometabolic risk factors should be encouraged among this group of professionals.

## Figures and Tables

**Table 1:** Haemodynamic, anthropometric, and dyslipidaemia atherogenic parameters of study population stratified by gender.

Parameter	Total 112 (100)	Female 64 (57.14)	Male 48 (42.86)	*p* value
Age	32.1 ± 8.9	32.3 ± 9.9	31.92 ± 7.5	0.8462
Age range				
22–30	68 (60.71)	43 (67.19)	25 (52.08)	0.0690
31–40	29 (25.89)	11 (17.19)	18 (37.50)	
41–50	6 (5.36)	3 (4.69)	3 (6.25)	
51–59	9 (8.04)	7 (10.94)	2 (4.17)	
SBP	117.7 ± 14.5	117.2 ± 16.4	118.3 ± 11.7	0.6813
DBP	76.7 ± 9.8	76.6 ± 10.3	76.9 ± 9.3	0.8664
BMI	25.3 ± 4.1	26.4 ± 4.4	23.9 ± 3.3	0.0019
TCh	5.4 ± 1.1	5.6 ± 1.1	5.2 ± 1.2	0.0867
TG	0.8 (0.3–3.8)	0.8 (0.3–3.2)	0.9 (0.4–3.8)	0.292
VLDL	0.4 (0.2–1.7)	0.4 (0.2–1.4)	0.4 (0.2–1.7)	0.288
FBS	4.7 ± 1.2	4.8 ± 1.5	4.6 ± 0.7	0.4404

Data are presented as mean ± standard deviation, median (minimum-maximum) or frequency (percentage). SBP: systolic blood pressure, DBP: diastolic blood pressure, BMI: body mass index, TCh: total cholesterol, TG: triglyceride, VLDL: very low-density lipoprotein, and FBS: fasting blood sugar. *p* is significant at 0.05.

**Table 2:** Prevalence of hypertension and haemodynamic presentation of health workers in Sefwi-Wiawso Municipal Hospital stratified by gender.

Parameter	Total	Female	Male	*p* value
*Normal*	35 (31.25)	21 (32.81)	14 (29.17)	0.0381
(SBP < 120 and DBP < 80)				
*Prehypertension*	59 (52.68)	36 (56.25)	23 (47.92)	
(SBP 120–139 or DBP 80–89)				
*Stage 1 hypertension*	15 (13.40)	4 (6.25)	11 (22.90)	
(SBP 140–159 or DBP 90–99)				
*Stage 2 hypertension*	3 (2.70)	3 (4.70)	0 (0.00)	
(SBP ≥ 160 or DBP ≥ 100)				
*Hypertension*	18 (16.07)	7 (10.94)	11 (22.92)	0.1191
(SBP ≥ 140 or DBP ≥ 90)				
*Hypertension with both SBP & DBP*	8 (7.14)	4 (6.25)	4 (8.33)	0.7227
(SBP ≥ 140 and DBP ≥ 90)				
*Hypertension with high SBP*	9 (8.04)	4 (6.25)	5 (10.42)	0.494
(SBP ≥ 140)				
*Hypertension with high DBP*	17 (15.18)	7 (10.94)	10 (20.83)	0.1197
(DBP ≥ 90)				
*Hypertension with isolated SBP*	1 (0.90)	0 (0.00)	1 (2.13)	0.4234
(SBP ≥ 140 and DBP < 90)				
*Hypertension with isolated DBP*	9 (8.11)	3 (4.69)	6 (12.77)	0.1645
(SBP < 140 and DBP ≥ 90)				

Data are presented as the frequency with the corresponding percentage in parenthesis. *p* is significant at 0.05. SBP: systolic blood pressure and DBP: diastolic blood pressure.

**Table 3:** Prevalence of cardiometabolic risk factors among health workers in Sefwi-Wiawso Municipal Hospital stratified by gender.

Parameter	Total	Female	Male	*p* value
Total respondent	112 (100)	64 (57.14)	48 (42.86)	
*Obesity classification*				
Underweight	2 (1.79)	1 (1.56)	1 (2.08)	0.0481
Normal	53 (47.32)	25 (39.06)	28 (58.33)	
Overweight	43 (38.39)	25 (39.06)	18 (37.50)	
Obesity class one	11 (9.82)	10 (15.63)	1 (2.08)	
Obesity class two	3 (2.68)	3 (4.69)	0 (0.00)	
Obesity	14 (12.50)	13 (20.31)	1 (2.08)	0.0037
*Atherogenic indices*				
Raised TCh	21 (18.75)	12 (18.75)	9 (18.75)	1
Raised TG	12 (10.71)	7 (10.94)	5 (10.42)	1
Raised VLDL	4 (3.57)	3 (4.69)	1 (2.03)	0.6339
*Atherogenic scores*				
None	82 (73.21)	48 (75.00)	34 (70.83)	0.3706
One	25 (22.32)	12 (18.75)	13 (27.08)	
Two	3 (2.68)	2 (3.13)	1 (2.08)	
Three	2 (1.79)	2 (3.13)	0 (0.00)	
*Glycaemia classification*				
Normoglycaemic	101 (90.18)	58 (90.63)	43 (89.58)	0.2925
Prediabetic	6 (5.36)	2 (3.13)	4 (8.51)	
Diabetes	5 (4.46)	4 (6.25)	1 (2.08)	

Data are presented as the frequency with the corresponding percentage in parenthesis. *p* is significant at 0.05. TCh: total cholesterol, TG: triglyceride, VLDL: very low-density lipoprotein, and FBS: fasting blood sugar.

**Table 4:** Age distribution of cardiovascular risks factors among health workers in Sefwi-Wiawso Municipal Hospital.

Parameter	Frequency	Percentage	Rank
*Hypertension*			
22–30	9	12.86	3rd
31–40	4	13.79	2nd
41–50	2	33.33	1St
51–59	3	33.33	1St
*Diabetes*			
22–30	2	2.86	3rd
31–40	0	0.00	4th
41–50	2	33.33	1St
51–59	1	11.11	2nd
*Obesity*			
22–30	5	7.14	4th
31–40	6	20.69	2nd
41–50	1	16.67	3rd
51–59	2	22.22	1St
*Dyslipidaemia*			
22–30	11	15.71	4th
31–40	11	37.93	1St
41–50	1	16.67	3rd
51–59	2	22.22	2nd

Data is presented as frequency, percentage, and ranks.

**Table 5:** Pearson bivariate correlation of cardiometabolic risk factors among hospital staff at Sefwi-Wiawso Municipal Hospital.

Parameter	BMI	SBP	DBP	FBS	TCH	TG	VLDL
Age	0.15	0.36^*∗∗*^	0.29^*∗∗*^	0.26^*∗∗*^	−0.01	0.28^*∗∗*^	0.28^*∗∗*^
BMI		0.17	0.16	0.21^*∗*^	0.28^*∗∗*^	0.24^*∗*^	0.24^*∗*^
SBP			0.74^*∗∗*^	0.37^*∗∗*^	0.04	0.08	0.08
DBP				0.17	0.00	0.01	0.00
FBS					0.19^*∗*^	0.32^*∗∗*^	0.31^*∗∗*^
TCH						0.28^*∗∗*^	0.27^*∗∗*^
TG							1^*∗∗*^

Data are presented as correlation coefficient of correlation. ^*∗*^*p* is significant at 0.05, ^*∗∗*^*p* is significant at 0.01, BMI: body mass index, SBP: systolic blood pressure, DBP: diastolic blood pressure, FBS: fasting blood sugar, TCh: total cholesterol, TG: triglyceride, and VLDL: very low-density lipoprotein.
